# Clinical predictors for deep vein thrombosis on admission in patients with intertrochanteric fractures: a retrospective study

**DOI:** 10.1186/s12891-021-04196-7

**Published:** 2021-04-02

**Authors:** Jixing Fan, Fang Zhou, Xiangyu Xu, Zhishan Zhang, Yun Tian, Hongquan Ji, Yan Guo, Yang Lv, Zhongwei Yang, Guojin Hou

**Affiliations:** Department of Orthopedics, Peking University Third Hospital, No. 49, North Garden Rd., Haidian District, Beijing, 100191 China

**Keywords:** Intertrochanteric fracture, Deep vein thrombosis, Admission, Risk factors, Elderly

## Abstract

**Background:**

Limited studies were available to investigate the prevalence of deep vein thrombosis (DVT) on admission in elderly patients with intertrochanteric fractures. The aim of present study was to evaluate risk factors and the prevalence of pre-admission DVT in elderly patients with intertrochanteric fractures.

**Methods:**

This retrospective study included 788 elderly patients with intertrochanteric fracture who were eligible for this study from January 1, 2010, to December 31, 2019. Color doppler ultrasonography was performed for DVT detection at admission. All patients’ clinical data were collected. Univariate analysis and stepwise backward multivariate logistic regression were used to identify the risk factors contributing to the occurrence of DVT.

**Results:**

The overall prevalence of pre-admission DVT in patients with intertrochanteric fractures was 20.81% (164 of 788 patients). The mean time from injury to admission was 2.1 days in the total population, 2.96 and 1.87 days in patients with and without DVT. Univariate analysis showed that significantly elevated risk of DVT were found in patients with longer time from injury to admission, high energy injury, lower Hb value, higher BMI, diabetes, chronic obstructive pulmonary disease (COPD), atrial fibrillation, dementia, varicose veins, higher age-adjusted CCI, higher ASA class and A3 type intertrochanteric fractures (*P* < 0.05). The adjusted multivariate logistic regression analysis demonstrated that longer time from injury to admission, high energy trauma, COPD, lower Hb, diabetes and A3 type intertrochanteric fractures were independent risk factors of pre-admission DVT.

**Conclusions:**

A high prevalence of pre-admission DVT was found in elderly Chinese patients with intertrochanteric fractures. Therefore, surgeons should be aware of the high prevalence of DVT for elderly patients with intertrochanteric fractures in order to prevent intraoperative and postoperative PE and other lethal complications.

## Background

Hip fracture is very frequent in the elderly, which is associated with increased disability and mortality [1]. It is estimated that the number of hip fractures is approximately 1.7 million each year, and the number is expected to surpass 6 million by the year 2050 worldwide [2]. When a hip fracture occurs, the risk of venous thromboembolism (VTE) increases obviously in the preoperative period owing to the venous congestion, vascular injury and immobilization [3]. Deep vein thrombosis (DVT) is one of the common complications in patients with hip fractures. Furthermore, the shed-off embolus can cause pulmonary embolism (PE), which remains the third most common cause of death in patients surviving the first 24 h of trauma [4]. Previous studies have shown that the incidence of preoperative DVT in hip fracture patients varied from 2.6 to 17.3%, and could be as high as 62% particularly in the hip fracture patients who had delayed operation [5, 6].

Furthermore, hip fracture patients are generally older with multiple medical comorbidities. Previous studies have shown that hip fracture patients coupled with medical comorbidities were predisposed to develop DVT [5, 7]. However, no appropriate tool has been well-established to quantify comorbidities for assessing the association between comorbidity and DVT. The Charlson Comorbidity Index (CCI) is an index of 19 conditions that are individually weighted based on the strength of their association with mortality, with the final score out of a possible 33 estimating the probability of death at 1 year [8]. However, rare studies have evaluated the association between CCI and DVT in hip fracture patients. In present study, we aim to assess the relationship between CCI and DVT in the elderly with hip fracture. Moreover, assessing medical comorbidities could prolong the preoperative waiting time, which is an important factor contributing to develop DVT in hip fracture patients.

Hip fractures are anatomically classified as intracapsular fractures (i.e., at the femoral neck) or extracapsular fractures (i.e., intertrochanteric or subtrochanteric fractures) in relation to the hip capsule with the similar frequency [9]. For anatomic reasons, extracapsular fractures tend to have a greater preoperative hemoglobin (Hgb) drop than intracapsular fractures [10]. In addition, preoperative anemia is a risk factor for preoperative DVT [11]. To the best of our knowledge, most of previous studies evaluating risk factors for DVT consisted of both femoral neck and trochanteric fractures. Currently, limited studies are available to investigate the prevalence of pre-admission DVT in elderly patients with intertrochanteric fractures. Therefore, the purpose of this study is to identify the prevalence of and risk factors for pre-admission DVT in patients with intertrochanteric fractures.

## Methods

This retrospective study was performed at a level 1 trauma center over a 10-year period from January 1, 2010, to December 31, 2019. Eight hundred seventy-two consecutive patients were diagnosed with intertrochanteric fractures at our hospital. Exclusion criteria for this study included age < 60 years, multi-type of fracture, secondary fracture, anticoagulant treatments, old fracture (>7d), incomplete medical records and other miscellaneous reasons. Of these 872 patients enrolled, 29 were younger than 60 years, 15 were old fractures, 8 were secondary fractures, 6 had multiple fractures and 26 cases had incomplete medical records. Overall, 84 patients were excluded from this study. Finally, a total of 788 elderly intertrochanteric fracture patients were included. This retrospective study was approved by the institutional ethical review board of our institution (Peking University Third Hospital, Beijing, China).

Patients were diagnosed with DVT by color Doppler ultrasonography, which was conducted by experienced radiologists in a color ultrasonic room. All ultrasound results were reviewed by a senior radiologist. When different opinions occurred, re-examination of the ultrasound results would be carried out by another senior radiologist. DVTs were classified into three types: central type, peripheral type, and mixed type. Thrombus occurred proximal to the knee in the iliacs, superficial femoral and/or femoral veins was regarded as central type. Thrombus occurred distal to the knee in the posterior tibial veins or peroneal veins was defined as peripheral type. DVT was classified as mixed type when involving the whole deep venous system of lower limb [11]. The diagnosis of DVT was according to the Robinov criterion, which included the following four parts: 1. In constant filling defects, thrombi were constant in appearance, and tended to be sharply delineated; 2. Abrupt termination of the opaque column occured at a constant site in a vain, either above or below the obstruction; 3. Nonfilling of the entire deep system or portions thereof when proper technique was used was abnormal and usually due to phlebitis; 4. Diversion of flow, representing collateral flow, was the counterpart if the nonfilling described above [12]. All patients wore compression stockings and underwent intermittent pneumatic compression from the time of admission except during external fixation, skeletal traction, or casting or if there were contraindications to using these mechanical prophylactic devices. If a DVT or PE was identified, a therapeutic dose of enoxaparin (100 IU/kg) was administered twice daily before surgery regardless of symptoms. Furthermore, the inferior vena cava filter placement was performed before operation when necessary. The criteria for inferior vena cava filter placement in our institution are as the following: the patient with thrombus/thrombi at the popliteal vein or above part, and the patient with absolute contraindications to therapeutic anticoagulation. Forty mg of enoxaparin was administered subcutaneously once daily beginning 2 days after surgery, and continued until hospital discharge. For patients with preoperative VTE, an initial 15 mg dose of rivaroxaban was administered orally twice a day for the first 21 days and a maintenance 20 mg dose was administered orally once a day for the remaining treatment duration.

All data were collected from the electronic medical records. Collected data included demographic variables (age, gender, BMI, injury side, injury mechanism, and time from injury to admission), hemoglobin value, comorbidities (including hypertension, diabetes, chronic obstructive pulmonary disease, atrial fibrillation, coronary heart disease, cerebrovascular accident, solid cancer, dementia, liver and kidney disease, thyroid disease, varicose veins), age-adjusted CCI, ASA classification, AO classification of intertrochanteric fractures. A high-energy injury was defined as an injury where there was a high possibility that multiple organs might be damaged due to mechanisms such as falling more than 4 ft, traffic accident, and direct blow. A low-energy injury was defined as an injury which patients would sustain while falling over slippery ground in a walking or sitting position.

### Statistical analysis

For quantitative data, the one-sample Kolmogorov-Smirnov test was used to test the normal distribution. The Student t-test or the Mann–Whitney test was used to compare continuous variables as appropriate. For qualitative data, the Chi-square test was used. When the factor’s *P* values was < 0.1, a multivariate logistic regression analysis was then performed to examine the association between possible risk factors and DVT. The percentage of patients with DVT for each day was evaluated to assess the DVT risk and the median time from injury to admission. A *P* value < 0.05 was considered statistically significant, and all tests were two-sided. SPSS 21.0 software was used for statistical analysis (SPSS, Chicago, Illinois, USA).

## Results

### Patient characteristics

A total of 788 elderly intertrochanteric fracture patients were included in this retrospective study. The patient’s age ranged from 60 years old to 113 years old and the mean age was 78.68(±7.89) years old. Of these patients, 273 (34.6%) were male patients and 515 (65.4%) were female patients. Of these 788 patients, 416 had left side injury and the other 372 had right side injury. Among these patients, 716 intertrochanteric fractures were caused by low energy injury and the left 72 cases were injured by high energy trauma. Of these patients, 174 had A1 type intertrochanteric fractures, 522 had A2 type intertrochanteric fractures, and 92 had A3 type intertrochanteric fractures. Of these intertrochanteric fractures, 28 cases were treated conservatively, 1 patient was treated by hemiarthroplasty, 664 patients were treated by intramedullary fixation, and the left 95 patients were treated by extramedullary fixation. Of these patients, 48 patients underwent inferior vena cava filter placement before operation.

### Prevalence of pre-admission DVT

The overall prevalence of pre-admission DVT in patients with intertrochanteric fractures was 20.81% (164 of 788 patients). Of these 164 patients with DVT, 9 (5.49%) had central DVTs, 153 (93.29%) had peripheral DVTs, and 3 (1.83%) had mixed DVTs. Three patients with DVT developed dyspnoea, tachypnoea, and chest pain, and were diagnosed with pulmonary embolism (PE) by CT pulmonary angiogram (CTPA). One patients suffered from the PE during the operation, another patient suffered from the PE after admission 2 days and the left one suffered from the PE after surgery 1 day. After thrombolytic therapy, thrombus in pulmonary arteries proved to dissolve by CTPA.

### Univariate analysis of pre-admission DVT

**Fig. 1:**
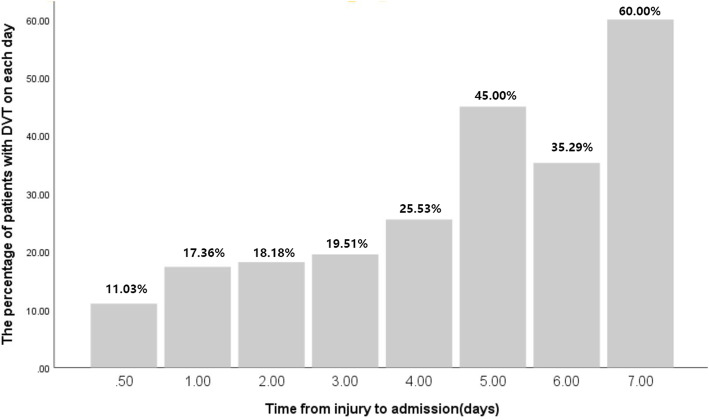
The percentage of patients with DVT for each day from injury to admission. DVT = deep vein thrombosis

In univariate analysis, the patients were divided into two groups: with and without DVT. The mean time from injury to admission was 2.1 days in the total population (Table 1). The mean time from injury to admission was 2.96 days for patients who developed DVT compared with 1.87 days for patients who didn’t develop DVT, and statistical difference was found significantly between two groups (*P* < 0.001). Furthermore, the risk of DVT increased significantly with a delayed admission (Fig. 1).

**Table 1:** Demographic characteristics and risk factors associated with pre-admission DVT

Variables	All patients (*n* = 788)	Patients without DVT(*n* = 624)	Patients with DVT (*n* = 164)	*P* value
Age (mean years±SD)	78.68 ± 7.89	78.49 ± 8.00	79.38 ± 7.44	0.199
Gender (Male/Female)				0.07
Male	273 (34.6%)	226 (36.2%)	47 (28.7%)	
Female	515 (65.4%)	398 (63.8%)	117 (71.3%)	
BMI (kg/m2)				0.005*
< 30	756 (95.9%)	605 (97.0%)	151 (92.1%)	
> 30	32 (4.1%)	19 (3.0%)	13 (7.9%)	
Injury side (left/right)				0.327
Left	416 (52.8%)	335 (53.7%)	81 (49.4%)	
Right	372 (47.2%)	289 (46.3%)	83 (50.6%)	
Injury mechanism				0.002*
Low energy trauma	716 (90.9%)	577 (92.5%)	139 (84.8%)	
High energy trauma	72 (9.1%)	47 (7.5%)	25 (15.2%)	
Time from injury to admission (days)	2.10 ± 1.79	1.87 ± 1.59	2.96 ± 2.22	< 0.001†
Hemoglobin(g/L)	113.92 ± 19.09	115.45 ± 18.93	108.13 ± 18.62	< 0.001†
Comorbidities				
Hypertension	440 (55.8%)	350 (56.1%)	90 (54.9%)	0.781
Diabetes	238 (30.2%)	171 (27.4%)	67 (40.9%)	0.001*
COPD	87 (11.0%)	54 (8.7%)	33 (20.1%)	< 0.001*
Atrial fibrillation	60 (7.6%)	34 (5.4%)	26 (15.9%)	< 0.001*
Coronary heart disease	162 (20.6%)	121 (19.4%)	41 (25.0%)	0.114
Cerebrovascular accident	172 (21.8%)	128 (20.5%)	44 (26.8%)	0.081
Cancer	78 (9.9%)	65 (10.4%)	13 (7.9%)	0.342
Dementia	35 (4.4%)	21 (3.4%)	14 (8.5%)	0.004*
Liver and kidney disease	43 (5.5%)	35 (5.6%)	8 (4.9%)	0.714
Thyroid disease	27 (3.4%)	18 (2.9%)	9 (5.5%)	0.103
Varicose veins	46 (5.8%)	29 (4.7%)	17 (10.4%)	0.005*
Age-adjusted CCI	4.27 ± 1.44	4.17 ± 1.42	4.63 ± 1.44	< 0.001†
ASA class				0.001*
1	26 (3.3%)	24 (3.8%)	2 (1.2%)	
2	576 (73.1%)	470 (75.3%)	106 (64.6%)	
3	180 (22.8%)	127 (20.4%)	53 (32.3%)	
4	6 (0.8%)	3 (0.5%)	3 (1.8%)	
AO classification				0.008*
A1	174 (22.1%)	145 (23.2%)	29 (17.7%)	
A2	522 (66.2%)	417 (66.8%)	105 (64.0%)	
A3	92 (11.7%)	62 (9.9%)	30 (18.3%)	

*Abbreviations*: *DVT* Deep vein thrombosis, *BMI* Body mass index, *COPD* Chronic obstructive pulmonary disease, *CCI* Charlson Comorbidity Index, *ASA* American Society of Anaesthesiologists, *AO* Arbeitsgemeinschaft fur Osteosynthesefragen

*Significant difference between two groups as shown by the Chi-square test; †Significant difference between two groups as shown by the Student t-test or the Mann–Whitney test. The values were given as the mean and standard deviation

In the DVT group, there was a higher prevalence of high energy injury (15.2% versus 7.5%, *P* = 0.002). In addition, patients in DVT group tended to have a higher prevalence of BMI > 30 kg/m2 than patients in non-DVT group (7.9% versus 3.0%, *P* = 0.005). With respect to comorbidities, patients with DVT on admission had a higher prevalence of diabetes (40.9% versus 27.4%, *P* = 0.001), chronic obstructive pulmonary disease (20.1% versus 8.7%, *P* < 0.001), atrial fibrillation (15.9% versus 5.4%, *P* < 0.001), dementia (8.5% versus 3.4%, *P* = 0.004) and varicose veins (10.4% versus 4.7%, *P* = 0.005). Furthermore, patients with DVT had a higher age-adjusted CCI than patients without DVT (4.63 versus 4.17, *P* < 0.001). Patients with DVT tended to have a lower level of hemoglobin (115 versus 108, *P* < 0.001). Statistically significant differences were found in terms of ASA class and AO classification between patients with and without DVT on admission (*P* = 0.001 and 0.008, respectively). However, no statistically significant differences were observed in age, gender, injury side, hypertension, coronary heart disease, cerebrovascular accident, cancer, liver and kidney disease and thyroid disease (*P* > 0.05).

### Multivariate analysis of pre-admission DVT

The results of the adjusted multivariate logistic regression analyses were presented in Table 2. There were significant associations between age-adjusted CCI, ASA classification and DVT on admission in the univariate analysis. The age-adjusted CCI, and ASA classification were no longer significant predictors of DVT after adjusting for potential confounders, because the age-adjusted CCI and ASA classification were highly associated with other risk factors. Furthermore, the BMI was no longer significant risk factor of DVT (OR = 2.022, 95%CI = 0.880–4.648, *P* = 0.097). After adjustment for other risk factors, the results showed that female sex was a significant risk factor for DVT (OR = 1.634, 95%CI = 1.057–2.527, *P* = 0.027) compared with male sex. A higher hemoglobin value (OR = 0.975, 95%CI =0.965–0.986, *P* < 0.001) would decrease the risk of DVT. Moreover, high energy trauma (OR = 2.451, 95%CI = 1.348–4.458, *P* = 0.003) and longer time from injury to admission (OR = 1.377, 95%CI = 1.249–1.519, *P* < 0.001) were independent risk factors for DVT after adjusted multivariate logistic regression analyses. In addition, patients with chronic obstructive pulmonary disease (OR = 2.229, 95%CI =1.289–3.855, *P* = 0.004) and diabetes (OR = 1.866, 95%CI = 1.248–2.791, *P* = 0.002) had higher risk than patients without chronic obstructive pulmonary disease, and diabetes. Moreover, Patients with A3 intertrochanteric fractures (OR = 3.287, 95%CI = 1.700–6.355, *P* < 0.001) had a higher risk than patients with A1 and A2 intertrochanteric fractures.

**Table 2:** Multivariate logistic regression analysis for risk factors associated with pre-admission DVT

Risk factors	Adjusted Odds Ratio(95%CI)	*P* Value
Age (years)	1.014 (0.987–1.043)	0.304
Gender		
Male	1.0(reference)	
Female	1.634 (1.057–2.527)	0.027*
BMI (kg/m2)		
< 30	1.0(reference)	
> 30	2.022 (0.880–4.648)	0.097
Injury mechanism		
Low energy trauma	1.0(reference)	
High energy trauma	2.451 (1.348–4.458)	0.003*
Time from injury to admission (days)	1.377 (1.249–1.519)	< 0.001*
Hemoglobin(g/L)	0.975 (0.965–0.986)	< 0.001*
Comorbidities		
Diabetes	1.866 (1.248–2.791)	0.002*
COPD	2.229 (1.289–3.855)	0.004*
Atrial fibrillation	1.735 (0.935–3.217)	0.080
Cerebrovascular accident	1.304 (0.819–2.076)	0.263
Dementia	1.838 (0.825–4.096)	0.136
Varicose veins	1.802 (0.882–3.682)	0.106
Age-adjusted CCI	0.873 (0.717–1.062)	0.174
ASA classification		0.092
1	1.0(reference)	
2	3.130 (0.691–14.172)	0.139
3	4.835 (1.031–22.680)	0.046*
4	3.495 (0.333–36.682)	0.297
AO classification		
A1	1.0(reference)	
A2	1.303 (0.796–2.135)	0.293
A3	3.287 (1.700–6.355)	< 0.001*

The multivariable regression analysis used backward selection with use of the likelihood ratio test to assess significance

*Abbreviations*: *DVT* Deep vein thrombosis, *BMI* Body mass index, *COPD* Chronic obstructive pulmonary disease, *CCI* Charlson Comorbidity Index, *ASA* American Society of Anaesthesiologists, *AO* Arbeitsgemeinschaft fur Osteosynthesefragen, *CI* Confidence interval

*The difference was significant

## Discussion

Owing to the venous congestion, vascular injury and immobilization and other medical problems, patients with hip fractures had an increased risk of developing DVT [5]. Previous studies had investigated the prevalence and risk factors of preoperative DVT, but fewer studies assessed the prevalence of DVT on admission in elderly Chinese patients with intertrochanteric fractures [13–15]. In fact, many hip fracture patients might have already had DVT on admission. Therefore, early DVT identification is essential to limit late complications of DVT and prevent clot extension, acute PE, and recurrent thrombosis. Additionally, most of previous studies evaluating risk factors for DVT consisted of both femoral neck and trochanteric fractures. Unlike femoral neck fractures, trochanteric fractures tended to have a greater risk of preoperative hemoglobin (Hgb) drop, which is a risk factor for preoperative DVT [10, 11]. To our knowledge, this study provides the first evidence of risk factors for pre-admission DVT in elderly Chinese patients with intertrochanteric fractures.

In this study, the overall prevalence of pre-admission DVT was 20.81% in patients with intertrochanteric fractures, which was a relative high incidence of DVT compared with previous studies [5, 6]. A longer waiting time was one of the most important factors contributing to the high prevalence of preoperative DVT [6, 13]. The time from injury to surgery consisted of time waiting for admission and the period awaiting surgery for preoperative evaluation. The main reason for longer time from injury to admission was transfer from community hospitals to our trauma center and the poor comorbidity of elderly patients, which needed more time for preoperative preparation [16]. Ideally, surgery should be performed as early as possible for early mobilization and relieve of pain [17]. In present study, the mean time from injury to admission was significantly longer in patients who developed DVT compared with patients who didn’t develop DVT. Furthermore, an increasing linear association was found between the occurrence of DVT and the time from injury to admission on cumulative hazard plotting. One possible reason might be that prolonged immobilization could result in venous congestion. Another reason might be that the fracture could lead to vascular injury, which might activate the coagulation system. The third reason might that fracture was frequently coupled with dominant and hidden blood loss, especially hidden blood loss for intertrochanteric fractures. Therefore, earlier admission was necessary for intertrochanteric fractures.

In present study, patients in DVT group tended to have a higher prevalence of BMI > 30 kg/m2 than patients in non-DVT group. Patients with higher level of BMI had a two-fold risk of DVT; however, this difference was not significant. An increased BMI was associated with venous thromboembolism, with multiple mechanisms and pathways contributing to this effect. One study revealed that obesity was associated with DVT [18]. The possible mechanism might be that an increased BMI could not only alter the expression of proteins of the coagulation and fibrinolytic cascade, but also change the platelet biology and function, which could promote the increased thrombotic risk [19]. In addition, some studies reported that female patients had a higher risk of DVT than male patients, while others had different opinions [5, 15, 20]. Our study revealed that female sex was a risk factor for DVT in elderly intertrochanteric fractures. Moreover, this study demonstrated that the incidence of DVT on admission in patients with high-energy injury was significantly higher than in patients with low-energy injury (34.7% versus 19.4%, *P* = 0.002). The adjusted multivariate logistic regression analysis showed that high energy injury was an independent risk factor for DVT on admission in patients with intertrochanteric fractures, which was supported by a previous study [21].

Various studies had demonstrated that the occurrence of DVT in patients with hip fractures was strongly associated with medical comorbidities, especially in elderly patients [14]. However, which specific comorbidity related to the occurrence of DVT remained controversial in previous studies [5, 22]. Shin et al. [5] investigated the prevalence and risk factors of preoperative VTE in 208 patients with hip fractures and identified that pulmonary disease and VTE history were independent predictive factors for preoperative VTE. Another study reported that coronary heart disease was independent risk factor of DVT in patients with hip fractures [22]. The possible reason might be that coronary heart disease was associated with hypercoagulability state [23]. In present study, we identified that diabetes was independent risk factor for DVT on admission in patients with intertrochanteric fractures. Furthermore, we found that COPD was independent risk factor for DVT on admission. The possible reason might be that COPD was associated with an increased atherosclerotic disease burden derived from a chronic inflammation [24].

The CCI was first reported in 1987 to estimate the probability of death within 1 year [8]. The CCI also correlated with the probability of death for patients with breast cancer, and adverse events after spine surgery [25]. However, rare studies evaluated the association between CCI and the occurrence of DVT in hip fracture patients. In present study, the mean value of age-adjusted CCI in patients with DVT was significantly higher than in patients without DVT (4.63 versus 4.17, *P* < 0.001). However, the multivariate logistic regression analysis showed that the age-adjusted CCI was no longer significant predictor of DVT on admission after adjusting for potential confounders. The possible reason might be that the age-adjusted CCI was highly associated with other risk factors, especially age and medical comorbidities. This result was supported by one previous study, which reported that CCI was not related with the occurrence of preoperative DVT [14]. Till now, there was not scientific evaluation method to assess the relationship between preoperative comorbidities and the occurrence of DVT in patients with hip fractures. A larger, prospective and multi-center study was necessary to develop a scientific evaluation system.

In this study, we demonstrated that A3 type intertrochanteric fracture was independent risk factor for the occurrence of DVT on admission in patients with intertrochanteric fractures. Shin et al. [5] demonstrated that subtrochanteric fracture was independent predictive factor for preoperative VTE in patients with hip fracture whose surgery was delayed by > 24 h. For anatomic reasons, extracapsular fractures tended to have a greater blood loss than intracapsular fractures. It was known that the anemia and low hemoglobin concentrations were significantly associated with frailty [26]. Moreover, frailty had been demonstrated to predict adverse outcomes in older surgical patients [27]. Furthermore, one study demonstrated that preoperative anemia is a risk factor for preoperative DVT in hip fracture patients [11], and preoperative anemia was very common in patients with intertrochanteric fractures due to the dominant and hidden blood loss. In present study, patients with DVT tended to have a lower level of hemoglobin (115 versus 108, *P* < 0.001). Furthermore, a lower level of hemoglobin was independent risk factor of DVT after adjusted multivariate logistic regression analyses.

There were several limitations existed in this study. First, this study was a retrospective study. Second, all patients in this study came from one trauma center. Therefore, a multi-center large sample study would be required to validate our findings. Third, laboratory tests were not obtained in this study. Previous studies showed that increased D-dimer was independent risk factor for the occurrence of DVT [11, 15]. Fourth, patients might have a DVT before injury.

## Conclusions

In conclusion, a high prevalence of DVT on admission of elderly Chinese patients with intertrochanteric fractures was identified in present study. In addition, a longer waiting time for admission was one of the most important factors contributing to the occurrence of pre-admission DVT. We also identified that high energy injury, female sex, diabetes, COPD and A3 intertrochanteric fracture were independent risk factors of pre-admission DVT in patients with intertrochanteric fractures. Therefore, sufficient evaluation and proper thromboprophylaxis should be performed for patients with intertrochanteric fractures in order to prevent intraoperative and postoperative PE and other lethal complications.

## Data Availability

All data generated and analysed during this study are included in this article.

## Abbreviations

VTE: Venous thromboembolism
DVT: Deep vein thrombosis;
PE: Pulmonary embolism (PE)
CCI: Charlson Comorbidity Index
BMI: Body mass index
CI: Confidence interval
OR: Odds ratio
COPD: Chronic obstructive pulmonary disease
